# Priming of mononuclear cells with a combination of growth factors enhances wound healing *via* high angiogenic and engraftment capabilities

**DOI:** 10.1111/jcmm.12152

**Published:** 2013-10-09

**Authors:** Enze Jin, Jong-Min Kim, Sung-Whan Kim

**Affiliations:** a Department of Cardiology, The Fourth Affiliated Hospital of Harbin Medical UniversityHarbin, China; b Department of Anatomy and & Cell Biology and Mitochondria Hub Regulation Center, College of Medicine, Dong-A UniversityBusan, South Korea; c Regional Clinical Trial Center, Dong-A University HospitalBusan, South Korea

**Keywords:** growth factor, mononuclear cell, wound healing, transplantation

## Abstract

Recently, we demonstrated that a specific combination of growth factors enhances the survival, adhesion and angiogenic potential of mononuclear cells (MNCs). In this study, we sought to investigate the changes of the angiogenic potential of MNCs after short-time priming with a specific combination of growth factors. MNCs were isolated using density gradient centrifugation and incubated with a priming cocktail containing epidermal growth factor (EGF), insulin-like growth factor (IGF)-1, fibroblast growth factor (FGF)-2, FMS-like tyrosine kinase (Flt)-3L, Angiopoietin (Ang)-1, granulocyte chemotactic protein (GCP)-2 and thrombopoietin (TPO) (all 400 ng/ml) for 15, 30 and 60 min. Wounds in nonobese diabetic-severe combined immune deficiency (NOD-SCID) mice were created by skin excision followed by cell transplantation. We performed a qRT-PCR analysis on the growth factor–primed cells. The angiogenic factors vascular endothelial growth factor (VEGF)-A, FGF-2, hepatocyte growth factor (HGF), platelet-derived growth factor (PDGF) and interleukin (IL)-8 and the anti-apoptotic factors IGF-1 and transforming growth factor-β1 were significantly elevated in the MNCs primed for 30 min. (T30) compared with the non-primed MNCs (T0). The scratch wound assay revealed that T30- conditioned media (CM) significantly increased the rate of fibroblast-mediated wound closure compared with the rates from T0-CM and human umbilical vein endothelial cells (HUVEC)-CM at 20 hrs. *In vivo* wound healing results revealed that the T30-treated wounds demonstrated accelerated wound healing at days 7 and 14 compared with those treated with T0. The histological analyses demonstrated that the number of engrafted cells and transdifferentiated keratinocytes in the wounds were significantly higher in the T30-transplanted group than in the T0-transplanted group. In conclusion, this study suggests that short-term priming of MNCs with growth factors might be alternative therapeutic option for cell-based therapies.

## Introduction

The cutaneous wound healing process is a well-orchestrated molecular and biological event consisting of cell proliferation, angiogenesis, extracellular deposition and remodelling 1–2. For the treatment of wounds, one of the most important factors underlying the impaired healing process might be reduced levels of cytokine release from specific local cells 3. Specifically, patients with diabetes can easily experience impaired wound healing and severe outcome, such as a high amputation rate 4.

Currently, stem cell-based therapies have emerged as a novel and promising strategy for damaged tissue regeneration 5. However, cell therapy has been hindered by the low engraftment and subsequent marginal effectiveness of administered cells 6–7. This phenomenon might be associated with poor viability and low survival potential of such transplanted cells in ischaemic and harsh environments.

The differentiation of transplanted cells is one of the main strategies used in stem cell therapy. Bone marrow–derived mesenchymal stem cells developed keratinocyte phenotype when they were transplanted into the epidermis 8. Other investigators have also reported that circulating monocytes have plasticity 9,10 and specifically transdifferentiated into keratinocyte-like cells 12.

Recently, we demonstrated that a specific combination of growth factors enhances the survival, adhesion and angiogenic potential of mononuclear cells (MNCs) 13. However, short-term stimulation of MNCs with growth factors and their effects regarding angiogenic properties are not fully elucidated. In fact, to be used as a practical treatment, minimal cell manipulation is important, especially for the patient with acute status.

In this study, we investigated the possibility that short-term manipulation with a combination of growth factors can enhance angiogenic potential of MNCs and those cells can subsequently improve wound healing.

## Materials and methods

### Collection and priming of MNCs

Human peripheral blood was obtained from healthy donors and MNCs were isolated as previously described 14. Briefly, blood was diluted with an equal volume of a washing buffer containing the following: phosphate-buffered saline (PBS), pH 7.2, + 0.5% BSA + 2 mM EDTA. The MNCs were harvested from the interface and washed with a magnet-activated cell sorting (MACS) washing buffer (Miltenyi Biotec, Auburn, CA, USA). The diluted cell suspension was layered over Histopaque (Pharmacia, Uppsala, Sweden) and centrifuged at 800 × *g* for 30 min. The MNCs were harvested from the interface, washed with MACS buffer and incubated with a priming cocktail containing EGF, IGF, FGF-2, Flt-3L, Ang-1, GCP-2 and TPO (all at 400 ng/ml) for 30 min. The primed MNCs were washed with MACS washing buffer and centrifuged at 800 × *g* for 10 min. All protocols involving human samples were approved by the Dong-A University Institutional Review Board, and the experiments conform to the principles established in the Declaration of Helsinki.

### Real-time PCR analysis

Quantitative real-time (qRT-PCR) assays were performed as reported previously 15. Briefly, total RNA was isolated from MNCs with the RNA-stat reagent (Iso-Tex Diagnostics, Friendswood, TX, USA) according to the manufacturer’s instructions. The RNA was subsequently reverse-transcribed with Taqman Reverse Transcription Reagents (Applied Biosystems, Foster City, CA, USA) according to the manufacturer’s protocol. The synthesized cDNA was subjected to qRT-PCR with specific primers and probes, and the RNA levels were quantitatively measured with an ABI PRISM 7000 Sequence Detection System (Applied Biosystems). The relative mRNA expression was normalized to GAPDH expression and calculated as reported previously 15–16. All primer/probe sets were purchased from Applied Biosystems. The catalogue numbers of the probes were as follows: for human, VEGF-A (Hs99999070_m1), Ang-1 (Hs00181613_m1), HGF (Hs00300159_m1), FGF-2 (Hs00266645_m1), platelet-derived growth factor (PDGF; Hs00966526_m1), EGF (Hs01099999-m1), IGF-1 (Hs01547657-m1), transforming growth factor (TGF) -β1 (Hs00998133_m1), IL-8 (Hs00174103_m1) and GAPDH (Hs99999905-m1); for mouse, VEGF-A (Mm00437306_m1), FGF-2 (Mm01285715_m1) and GAPDH (Mm99999915_g1).

### Conditioned media (CM) preparation

Conditioned media was harvested as previously described 17. MNCs (1 × 10^7^ cells each) were seeded into T-75 cell culture flasks and grown in low-glucose DMEM (Gibco, Grand Island, NY, USA) containing 10% FBS, 100 U/ml penicillin and 100 μg/ml streptomycin (Gibco) for 7 days. The CM was then centrifuged at 800 × *g* for 15 min., and the supernatants were harvested and used in this assay. Human umbilical vein endothelial cells (HUVEC) were purchased from ATCC (Manassas, VA, USA). HUVEC-CM was used as control.

### Scratch wound assay

Human dermal fibroblasts (HDFs) were purchased from ATCC. The scratch wound assay was conducted as previously reported 18. Briefly, HDFs were seeded to a final density of 1 × 10^5^ cells/well in 24-well culture plates and incubated at 37°C in 5% CO_2_ to create confluent monolayers. The confluent monolayers were scratched with a sterile pipette tip and incubated with specific CM. To measure cell mobility, we took pictures from seven random fields at 5 and 20 hrs after scratching. The wound area was measured by the wound margin and calculated with the NIH Image program (http://rsb.info.nih.gov/nih-image/).

### Cell adhesion assays

Adhesion assays were conducted with modified, previously reported protocol 14–19. MNCs (2.5 × 10^4^/well) were seeded on 96-well plates pre-coated with 20 μg/well fibronectin (Sigma-Aldrich, St Louis, MO, USA) in EGM-2 medium for 24 hr at 37°C and 5% CO_2_. The cells were gently washed three times with PBS to remove the non-adherent cells, and adherent cells were enumerated by independent blinded investigators.

### Full-thickness excisional wound model and cell transplantation

Male NOD/SCID mice that were 12–13 weeks old and weighed 20–26 g were randomly divided into four groups as follows: sham (control, *n* = 7), HUVEC-injected (HUV, *n* = 7), T0-injected (T0, *n* = 7) and T30-injected groups (T30, *n* = 7). The excisional splinting model was performed as reported previously 18. For each mouse, the hair was removed from the dorsal surface, and then two 6-mm full-thickness excision skin wounds were created on each side of the midline. MNCs (1 × 10^7^ cells suspended in 100 μl PBS) labelled with chloromethylbenzamido-1,1′-dioctadecyl-3,3,3′3′-tetramethylindo-carbocyanine (CM-Dil; Molecular Probes, Eugene, OR, USA) were transplanted intradermally around the wound. In the control group, 100 μl of PBS was injected around the wounds. A doughnut-shaped silicone splint (Grace Bio-Labs, Bend, OR, USA) was used with the wounds centred within the splint. To fix the splint to the skin, an immediate bonding adhesive (Krazy Glue; Elmer’s Inc., Columbus, OH, USA) was used, and Tegaderm (3M; Health Care, St. Paul, MN, USA) was placed over the wounds. Experimental protocols were approved by the Dong-A University’s Institutional Animal Care and Use Committee, and all procedures were performed in accordance with the Guide for the Care and Use of Laboratory Animals published by the US National Institutes of Health (NIH Publication No. 85-23, revised 1996).

### Wound analysis

Wound analysis was performed by tracing the wound margin with a fine-resolution computer mouse and calculating the enclosed pixel area with NIH image software, image J (NIH Image, Bethesda, MD, USA) by investigators who were blinded to grouping and treatments, as previously described 18. The rate of wound closure was calculated as follows: original wound area − new wound area/original wound area × 100. The splint-generated holes represent the original wound size.

### Histological analysis

A histological analysis was performed as previously described 18. The mice were killed, and skin wound samples were harvested with a 10-mm biopsy punch. The tissues were fixed with 4% paraformaldehyde for 1 day and embedded using the OCT compound (Sakura Finetek USA, Torrance, CA, USA). For immunofluorescence studies, the tissue sections were incubated with sodium borohydride (1 mg/ml) to eliminate auto-fluorescence. Sections of each group were stained with the primary mouse anti-cytokeratin (1:200, Abcam, Cambridge, MA, USA) and the secondary Cy2 (1:400, Jackson ImmunoResearch, West Grove, PA, USA) antibodies. Nuclei were stained with DAPI (Sigma-Aldrich). Six fields from six tissue sections were randomly chosen, and the number of positive cells in the field was counted for the quantification of engrafted and differentiated cells. To evaluate apoptosis, the TdT-mediated dUTP nick-end labelling (TUNEL) reaction was conducted as previously reported using a fluorescein *in situ* cell death detection kit (Roche Molecular Biochemicals, Mannheim, Germany) 13.

### Statistical analysis

All data are presented as the mean ± SEM. Student’s *t*-test for comparing two groups and an anova with Bonferroni’s multiple comparison tests were performed with the SPSS v11.0 software (SPSS Inc., Chicago, IL, USA); *P* < 0.05 was considered statistically significant.

## Results

### Primed MNCs exhibit high angiogenic and anti-apoptotic properties

**Figure 1:**
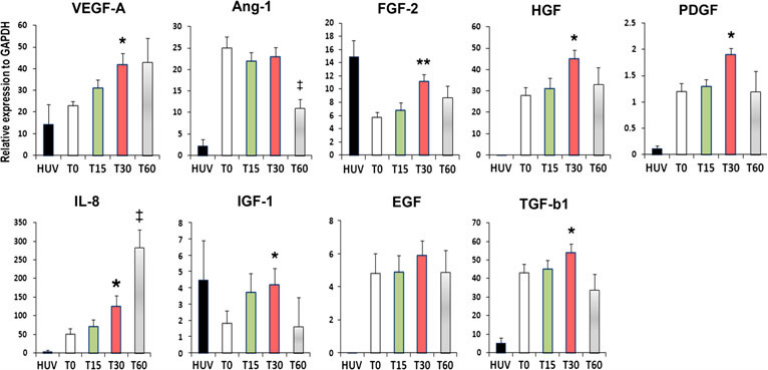
Angiogenic gene expression profile. The cells were harvested directly after 15, 30 and 60 min. after the priming. qRT-PCR was conducted to evaluate multiple anti-apoptotic gene expression levels in non-primed cells (T0), 15-minute (T15), 30-minute (T30) and 60-minute primed cells (T60). Multiple angiogenic genes were up-regulated in the T30 group when compared with T0 group. All individual values were normalized to GAPDH (*n* = 4 per group). HUV, HUVEC: human vein endothelial cells. **P < 0.01 T0 *versus* T30, *P < 0.05 T0 *versus* T30, ^‡^*P* < 0.01 T0 *versus* T60.

To measure the expression of multiple angiogenic and anti-apoptotic genes after MNC priming, a qRT-PCR analysis was performed. Angiogenic factors, VEGF-A, FGF-2, HGF and PDGF were significantly elevated in the 30-minute primed cells (T30) compared with the non-primed cells (T0; Fig. 1). One of the major angiogenic factors, chemokine, IL-8 (CXCL8), was significantly elevated in the T30 and T60 compared with the T0.

Other anti-apoptotic factors, IGF-1 (*P* < 0.05) and TGF-β1 (*P* < 0.05), were also significantly elevated in the T30 compared with the T0 (Fig. 1).

### Culture media (CM) from primed cells promotes wound closure and adhesion capacity

**Figure 2:**
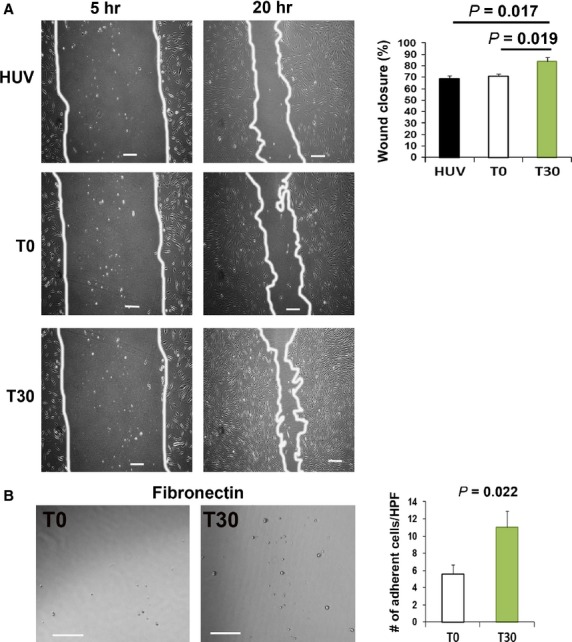
Scratch wound and cell adhesion assays. (A) Representative images of the scratch wound assay after incubation with CM. An *in vitro* wound healing assay revealed that T30-CM significantly improved fibroblast-mediated wound closure compared with the T0-CM and HUV-CM. (*n* = 4 per group). Bars: 200 μm. (B) Representative images of the result of adhesion assay. Cell adhesion assays demonstrated a higher adhesion capacity of T30 than T0 to fibronectin and the extracellular matrix (*n* = 4 per group). Bars: 100 μm. HPF: high power field.

To investigate whether proteins secreted from primed cells promote wound closure, we conducted a scratch wound assay. The scratch wound assay revealed that T30-CM significantly increased the rate of fibroblast-mediated wound closure compared with that from T0 and HUVEC-CM at 20 hrs (84.1 ± 2.7 *versus* 69.3 ± 2.7; *P* < 0.05 and 71.3 ± 1.8; respectively; *n* = 4; Fig. 2A). In addition, we examined the cell adhesion capacity because adhesion potential is an indicator of cell survival and engraftment in endothelial cells. The number of adhered cells to the extracellular matrix proteins fibronectin was significantly higher for the T30 cells than for the T0 cells (Fig. 2B).

### Primed cells enhance wound healing

**Figure 3:**
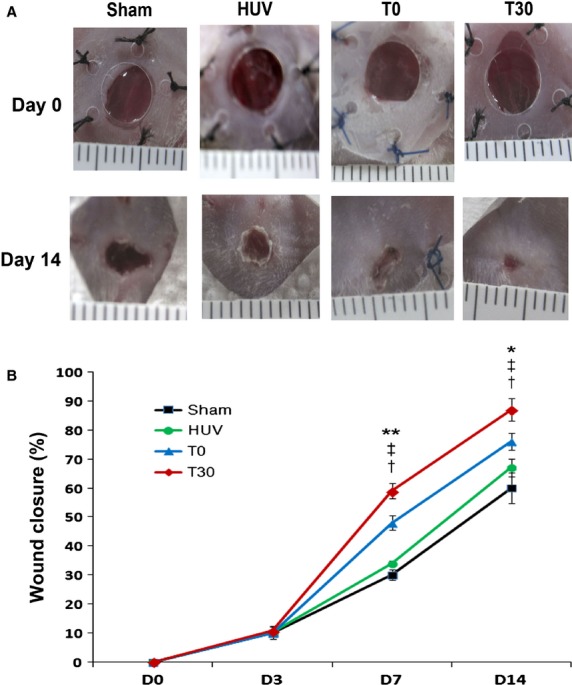
*In vivo* wound closure analysis. (A) Representative images of the excisional wound splinting mouse model after transplantations of control vehicle medium (sham), T0 and T30 at days 0, 3, 7 and 14. (B) Wound measurements of each group in NOD/SCID mice (*n* = 12 per group). **P < 0.01 T30 *versus* T0, *P < 0.05 T30 *versus* T0, ^‡^*P* < 0.01 T30 *versus* HUV, ^†^*P* < 0.01 T30 *versus* sham.

To examine the *in vivo* wound healing capacity of primed cells, we created excisional wounds with a NOD/SCID mouse model. T0, T30 and HUVEC were then injected into the dermis ∼0.4 cm from the wounds. Wound healing results revealed that T30-treated wounds demonstrated accelerated wound healing at days 7 and 14 compared with those treated with T0 (day 7: 58.8 ± 2.3% *versus* 48.2 ± 2.5%; *P* < 0.01, day 14: 87.3 ± 3.8% *versus* 76.3 ± 3.0%; *P* < 0.05; *n* = 12; Fig. 3A and B).

### Primed cells exhibit high engraftment and differentiation properties

**Figure 4:**
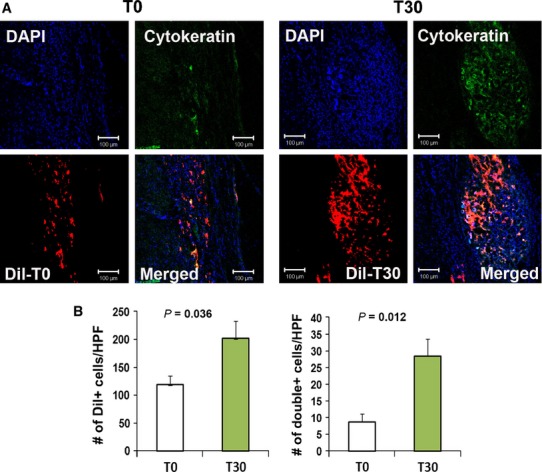
Engraftment capacity of T30 cells. (A) Representative Images of localized Dil-labelled T0 and T30 cells. (B) Quantification of engrafted T0 and T30 cells. Dil-labelled cells were injected into the peri-wound areas of NOD/SCID mice and the wound area tissues were harvested 4 weeks after cell transplantation. Transplanted Dil-labelled cells in the wound area were quantified using histological analysis. The nuclei (*n* = 7 per group) were stained with DAPI (blue).

To measure the engraftment of injected cells in skin wounds, we conducted immunohistochemistry on tissue specimens. The histological analysis demonstrated that the number of engrafted cells in wounds was significantly higher in the T30-transplanted group than in the T0-transplanted group (202 ± 30.7 *versus* 119 ± 15.7; *P* < 0.05, *n* = 5; Fig. 4A and B).

To investigate the keratinocyte differentiation capabilities of T30 and T0, we conducted immunostaining with the epithelial cell specific protein, cytokeratin. We identified some cells that transdifferentiated into keratinocytes in the epidermis near the wound areas. Four weeks after Dil-labelled cell implantation, higher numbers of cytokeratin and Dil double-positive stained cells were detected in the T30-treated wounds than in the T0-treated wounds (28.3 ± 5.2 *versus* 8.6 ± 2.0; *P* < 0.05, *n* = 7; Fig. 4A and B). These data suggest that the growth factor-primed cells possess an increased survival and keratinocyte differentiation potential in the treated wounds.

### Up-regulation of angiogenic factors and decreased apoptosis after T30 transplantation

**Figure 5:**
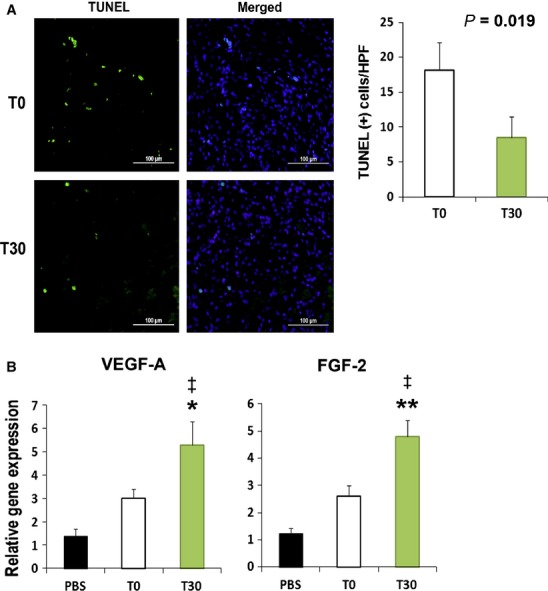
Apoptosis assay and angiogenic chemoattractant factors in skin wound tissues after cell transplantation. (A) Representative figures from TUNEL analysis. Quantification of TUNEL-positive (green fluorescence) nuclei after cell transplantation. The nuclei were stained with DAPI (blue fluorescence) in the skin wound at day 7. (*n* = 5 per group); bars: 100 μm. (B) Increased expression of angiogenic factors in tissues injected with T30. (*n* = 5 per group). **P < 0.01 T0 *versus* T30, *P < 0.05 T0 *versus* T30, ^‡^*P* < 0.01 PBS *versus* T30.

To investigate anti-apoptotic function, a TUNEL assay was conducted on tissue sections harvested on day 7 from the T30 and T0 treated skin wound tissues (Fig. 5A). The number of TUNEL-positive nuclei in the skin wound was approximately twofold lower in the T30 group than in the T0 group.

To define the mechanism responsible for the therapeutic effects, the gene expression levels of angiogenic factors were examined. The expression levels of VEGF-A and FGF-2 were higher, respectively, in T30-injected skin wound compared with T0-injected skin wound (Fig. 5B).

## Discussion

This study examined the angiogenic effects of short-term primed MNCs using a combination of growth factors. To our knowledge, we first demonstrated that short-term priming can enhance the angiogenic potential of MNC, as demonstrated by accelerated wound healing. Therefore, this approach might be an alternative therapeutic option for cell-based therapies.

Mononuclear cells are the most useful progenitor cell source for cellular therapy because it involves a less invasive, cell collection approach. However, the marginal therapeutic effect of these particular transplanted cells caused by low survival capacity and poor engraftment has become a major technical limitation. To enhance the efficacy of cell transplantation, a priming technique that improves the cellular cytoprotective effects and facilitates high engraftment of transplanted cells might be a rational approach. Many studies report a role of growth factors 13–20. Therefore, we hypothesized that a short-term priming approach with a combination of growth factors might enhance the angiogenic property of the MNCs. A rapid cell preparation is important for reducing time and cost and, specifically, for curing acute diseases.

Because our previous data demonstrated that a specific combination of growth factors, such as EGF, IGF-1, FGF-2, Flt-3L and TPO, affected the angiogenic, adhesion and survival properties of MNCs, we made a priming cocktail with those growth factors and incubated this cocktail with MNCs for 30 min. Traditionally, FGF-2, Flt-3L and TPO have been used to stimulate haematopoietic cell expansion. EGF and IGF-1 are reported to be associated with cell adhesion and anti-apoptotic functions 21–22. Ang-1, well-known angiogenic factor, can enhance collateral vascularization 23 and inhibit cell apoptosis *via* Akt signal pathway 24. GCP-2 also has been shown to exert a pro-angiogenic effect 25. Interestingly, the incubation of this growth factor priming cocktail, for even a short time period, significantly enhanced the expression of multiple angiogenic genes, such as VEGF-A, FGF-2, HGF, PDGF and IL-8. In addition, this priming cocktail also up-regulated the anti-apoptotic factors IGF-1 and TGF-β1. The augmented expression of FGF-2, HGF, PDGF and IGF-1 is consistent with the result of our previous cord blood–derived MNCs that were stimulated by growth factor combinations 13. These data indicate that the addition of high concentrations of growth factors within 30 min. can induce MNCs with enhanced angiogenic and survival properties.

During the wound healing process, cell migration is a key event. The main therapeutic mechanism of transplanted stem or progenitor cells is through paracrine effects; therefore, cell migration studies with secreted factors were performed. Consistent with the gene expression data, an *in vitro* scratch wound assay revealed that T30-CM markedly stimulated fibroblast cell migration when compared with the CM of T0 and HUVEC. These results indicate that priming for short time periods can stimulate MNCs to increase growth factor secretion levels. Real-time PCR data revealed that high IL-8 expression levels were observed in T30. Because IL-8 has been reported to promote cell proliferation and migration 26, enhanced IL-8 expression may be involved in cell migration.

The poor survival of transplanted stem/progenitor cells and their marginal therapeutic effects have become a hurdle to overcome for cell-based therapies. In this study, we observed that, in accordance with *in vitro* anti-apoptotic gene expression and adhesion assays, T30 showed a high capacity for engraftment, transdifferentiation and accelerated wound healing potential. The enhanced transdifferentiation of T30 into keratinocytes is one of the most prominent findings of this study. We speculate that some populations of the transplanted T30 transdifferentiated into keratinocytes and the combination of growth factors may stimulate and enhance this differentiation. However, we found that injected T-30 were stably engrafted in the epidermis, 4 weeks after cell injection. This finding implies that circulating monocytes have the potential to transdifferentiate into keratinocyte-like cells 12 and other types of cells 9–10, suggesting that the transdifferentiation of T-30 may contribute to their therapeutic effects. However, more cell differentiation studies, particularly long-term studies related to the fate and stability of transplanted cells, are required.

Autologous cell-based therapies using this cell-priming method for regenerative medicine can offer significant advantages, such as reducing operation times and labour.

In conclusion, this study demonstrated the beneficial effects of using growth factor-primed MNCs for wound healing through augmenting their paracrine and engraftment potential. Therefore, we propose that short-term cell priming can be an alternative method to enhance cell-based therapies.
